# The Role of Minimally Invasive Surgery in the Management of Inflammatory Bowel Disease: Current Trends and Future Directions

**DOI:** 10.7759/cureus.65868

**Published:** 2024-07-31

**Authors:** Sanskruti Rathod, Nishant Kumar, German D Matiz, Sheryl Biju, Peter Girgis, Nagma Sabu, Hassan Mumtaz, Ali Haider

**Affiliations:** 1 Surgery, Dr. Panjabrao Deshmukh Memorial Medical College, Amravati, IND; 2 Surgery, Christian Medical College, Vellore, IND; 3 Gastroenterology, Universidad El Bosque, Bogota, COL; 4 Medicine, Christian Medical College, Ludhiana, IND; 5 Internal Medicine, Ross University School of Medicine, Bridgetown, BRB; 6 Surgery, Jonelta Foundation School of Medicine, University of Perpetual Help System Dalta, Las Pinas City, PHL; 7 Urology, Guy's and St Thomas' Hospital, London, GBR; 8 Data Analytics, BPP University, London, GBR; 9 Allied Health Sciences, The University of Lahore Gujrat Campus, Gujrat, PAK

**Keywords:** public health care, improving health outcomes, robotic assited surgery, inflammatory bowel disease, minimally invasive surgery

## Abstract

Minimally invasive surgery (MIS) provides superior results in the surgical treatment of inflammatory bowel disease (IBD). There exist various minimally invasive procedures, each possessing its own set of benefits and drawbacks. This literature review outlines these methodologies and underscores their importance in enhancing the outcomes of patients with IBD. A grand total of 192 studies were carefully chosen and succinctly summarized. Conventional multiport laparoscopy is the most widely used MIS for IBD, with single-incision laparoscopy showing even better results. Robotic surgery offers comparable results but at higher costs and longer operation times. In the future, there will be widespread acceptance of single-incision laparoscopy and robotic surgery due to improved training and reduced expenses. Further research into the technology’s utility in different IBD presentations could increase its usage.

## Introduction and background

Inflammatory bowel disease (IBD) is a chronic inflammation of the digestive system, primarily characterized by ulcerative colitis (UC) and Crohn’s disease (CD). The exact cause is unknown, but factors include environmental, genetic, and gut microbiota [1]. Colonoscopy with biopsies is the gold standard for diagnosis because it can distinguish between the characteristic findings of UC and those of CD. 

UC is a chronic colon inflammation causing mucosal and submucosal ulcers. It’s caused by abnormal bacteria-immune interactions, causing increased permeability and proinflammatory cytokine secretion. Symptoms include bloody diarrhea, abdominal pain, and complications [2-4]. CD is a bimodal disease that causes inflammation, tissue damage, and fibrotic scarring in the digestive tract. It affects any part of the digestive tract and is associated with skip lesions. Patients with terminal ileum pain often present with lymphoid aggregates and granulomas [5-7]. In the 21st century, there has been a progressive incline in the incidence of IBD as a global disease in newly industrialized countries. The prevalence of IBD has surpassed 0.3% in Oceania, North America, and many countries in Europe [8]. A peak in the incidence of IBD is seen during adolescence or early adulthood, as almost one-quarter of all cases are diagnosed before 18 years of age [9-11]. 

IBD patients often require surgery due to the disease’s relapsing and remitting nature, with up to 30% of UC and 80% of CD requiring surgery. The ileal pouch-anal anastomosis (IPAA) is the favored procedure, with total proctocolectomy (the removal of the large intestine (the colon) and rectum, leaving the small intestine disconnected from the anus) being the preferred operation. Laparoscopic ileal pouch-anal procedure removes the colon and rectum while creating an internal pouch from a portion of a small intestine, which offers improved short-term outcomes and reduced hernia rates [12-16]. Surgery is recommended for refractory CD patients with severe complications. Common procedures include ileocecal resection, stricturoplasty, and endoscopic dilatation, which are treatments for strictures that can occur after ileocecal resection or right hemicolectomy. Recurrence rates vary, but surgical procedures improve morbidity, reduce trauma, and speed up recovery [17,18]. Minimally invasive surgery (MIS) can reduce the risk of intestinal obstruction, improve visualization, and decrease inflammation. Smaller wounds lead to less postoperative pain and morbidity. Robotic surgery offers advantages like motion scaling and 3D vision. While not all patients with IBD can undergo MIS, it may improve the management of IBD. However, contraindications may exist [19-22].

In this review, we will summarize the current trends in MIS in CD and UC.

## Review

Materials and methods

Literature Search Strategy

A comprehensive literature search was conducted using two electronic databases, MEDLINE/PubMed and Google Scholar, to identify studies published between January 2010 and December 2023 on the management of IBD using MIS. The following search terms were used: “Inflammatory Bowel Disease,” “Crohn’s Disease,” “Ulcerative Colitis,” “Minimally Invasive Surgery,” “Laparoscopy,” and “Laparoscopic.”

Inclusion and Exclusion Criteria

Studies were included if they met the following criteria:

-Study design: Primary research including observational studies, cohort studies, case-control studies, and randomized controlled trials.

-Focus: Studies specifically focused on the management of IBD using MIS.

-Language: Published in the English language.

-Time frame: Studies published between January 2010 and December 2023.

Studies were excluded if they were:

-Secondary research: Including narrative reviews, systematic reviews, and meta-analyses.

Data Extraction

Two groups of reviewers independently screened articles based on their titles and abstracts to identify relevant studies from PubMed and Google Scholar. The full texts of the relevant articles were then reviewed to ascertain whether they met the inclusion criteria. A summary table was created using data extracted from each included study, which included study design, sample size, demographics, intervention(s), and relevant outcomes.

Study Design

The included studies encompassed a range of primary research designs, such as observational studies, cohort studies, case-control studies, and randomized controlled trials. These study designs provided a comprehensive understanding of the management of IBD using MIS from various methodological perspectives. The selected time frame for these articles, spanning from January 2010 to December 2023, ensured the inclusion of recent and relevant research, capturing the latest advancements and trends in the field.

Data Synthesis and Summary

Data from the included studies were summarized after reviewing study design, patient characteristics, interventions, and outcomes. The results were presented in a descriptive manner according to the intervention type.

Results

In our literature review, we identified studies evaluating minimally invasive techniques for the surgical management of IBD, which were broadly classified into the following categories: conventional multiport laparoscopy, single-incision or single-port laparoscopy, and robotic or robot-assisted surgery. Below, we summarize the findings from these studies.

Role of MIS in IBD

In our literature review, we came across studies evaluating minimally invasive techniques for surgical management of IBD, which could be broadly classified into the following categories: conventional multiport laparoscopy, single-incision or single-port laparoscopy, and robotic or robot-assisted surgery. The results of studies comparing these approaches are discussed in this section. We also highlight the role of endoscopy and colonoscopy in the diagnosis, management, and surveillance of IBD.

Conventional Multiport Laparoscopy

The extensive body of literature on laparoscopic surgery for the management of IBD provides a comprehensive view of its efficacy and evolving trends. In the investigation of laparoscopic procedures for adult colorectal surgeons and adolescents with IBD, the safety of this combination is explored, offering insights into the feasibility and potential benefits for younger patients [23]. Long-term outcomes following laparoscopically assisted versus open ileocolic resection for CD contribute substantial evidence regarding the comparative effectiveness of laparoscopic techniques, providing a foundation for treatment decisions [24]. The feasibility of laparoscopic restorative proctocolectomy (LRP) without diverting stoma suggests the potential for minimizing invasive interventions, impacting the postoperative quality of life [25].

Adhesions after laparoscopic and open IPAA surgery for UC delineate the postoperative complications associated with different surgical approaches, aiding in refining surgical strategies [26]. The significantly increased pregnancy rates after LRP illuminate the broader impact of MIS on patients’ reproductive health [27].

Short-term outcomes of laparoscopy combined with enhanced recovery pathways shed light on the potential benefits of integrating laparoscopic techniques with enhanced recovery protocols, emphasizing holistic patient care [28]. The study comparing laparoscopic-assisted versus conventional ileocolectomy for primary CD delineates the nuances between these approaches, influencing decision-making in primary CD cases [29].

Preoperative infliximab therapy’s impact on morbidity and mortality after laparoscopic resection for IBD addresses a critical aspect of preoperative management, contributing to a nuanced understanding of the intersection between medical and surgical therapies. The extensive collection of articles underscores the dynamic landscape of laparoscopic surgery for IBD, from procedural nuances to technological advancements. Future trends may involve further integration of robotic assistance, personalized approaches based on patient characteristics, and continued refinement of laparoscopic techniques, as included in the study by Miller et al. on robotic-assisted proctectomy for IBD, which presents a case-matched comparison of laparoscopic and robotic techniques, assessing the role of advanced technologies in improving surgical outcomes [30,31]. As the field evolves, ongoing research will likely explore novel strategies to enhance patient outcomes, reduce complications, and broaden the scope of minimally invasive interventions in the realm of IBD surgery. 

Single-Incision or Single-Port Laparoscopy

Single-incision laparoscopy theoretically should offer faster recovery and reduced postoperative pain. There are several studies highlighting multiple benefits of single-incision laparoscopy over conventional laparoscopy for patients with CD, such as reduced hospital stay, reduced postoperative pain scores, and decreased need for analgesia post-surgery [32-34]. The rate of postoperative complications was similar in both groups in these studies. For patients with UC undergoing single-stage proctectomy with single-port laparoscopy, a study done by Li et al. reveals lower estimated blood loss, shorter operative time, and reduced length of stay in the hospital compared with multi-port laparoscopy [35]. Similar results were achieved by Burke et al. [36].

Robotic or Robot-Assisted Surgery

Robotic surgery has been the latest addition to the minimally invasive techniques used in the surgical management of IBD. When compared to the open approach, robot-assisted ileocolic resection for CD was associated with a shorter stay in the hospital and a lower rate of complication within 30 days of the surgery but longer operative times, as demonstrated by Raskin et al. [37]. However, for complex operations like IPAA, robot-assisted surgery offers no advantage over the open approach while incurring higher operative times, as highlighted by Opoku et al. [38]. Robotic surgery offers almost similar postoperative outcomes, including rates of complication, reoperation, and readmission, as compared to conventional laparoscopy, although with higher operative time [39-41]. 

When outcomes like return to bowel function, length of stay, and estimated blood loss are considered, its advantage over conventional laparoscopy remains unclear, with some studies favoring the robotic approach and some other studies favoring laparoscopy [39-43]. There is a need for a larger study, such as a randomized trial or systematic review with meta-analysis, to get conclusive evidence regarding its disadvantages and advantages over conventional laparoscopy. Moreover, when compared to single-incision laparoscopy, robotic surgery was found to have inferior results with increased estimated blood loss and longer length of stay, according to a study done by Rencuzogullari et al. [44].

There have also been concerns regarding the higher cost of robotic surgery without any clinically significant benefit over conventional laparoscopic surgery, as highlighted by Gebhardt et al. [45]. Advancements in technology and widespread adoption with increased surgeon experience may help reduce costs and operation times. 

Role of MIS in Pediatric Population

Minimally invasive techniques gain even more importance in the pediatric population considering the psychological impact of the length of the scar as well the overall quality of life after surgery in children. In our literature review, we found several studies that have highlighted that laparoscopic surgery has better outcomes in terms of lower complication rate and shorter length of stay compared to the open approach in children with UC [46,47]. 

Similar results were obtained by Quiroz et al. in a study done on children with CD [48]. Studies have also been done on single-incision laparoscopic surgery (SILS) and robot-assisted surgery in children with IBD, and all have highlighted these techniques as a safe and feasible option, but studies comparing these techniques in children with conventional laparoscopy have not yet been done [49-51]. 

Role of Endoscopy

The comprehensive review of literature on endoscopic interventions within MIS for IBD reveals a multifaceted landscape of evolving approaches and promising outcomes. Laparoendoscopic single-site surgery emerges as a feasible alternative for complex colorectal resections, presenting a potential paradigm shift by enabling day case colectomy [52]. The application of surgical stricturoplasty in treating ileal pouch strictures provides insights into tailored interventions, offering an alternative to conventional surgical methods [53]. Innovative avenues in postoperative care are explored in a study by Fedorak et al., where the probiotic VSL#3 exhibits anti-inflammatory effects, potentially reducing endoscopic recurrence after CD surgery [54].

The intricate relationship between prior use of immunomodulatory drugs and clinical outcomes of endoscopic balloon dilation for intestinal strictures in CD patients is elucidated in the article by Honzawa et al. [55]. A notable contribution comes from a large UK series, as discussed in an article by Karahan and Sevinç, demonstrating the safety and efficacy of endoscopic balloon dilatation for CD strictures [56]. Additionally, the long-term outcomes of serial endoscopic balloon dilation for upper gastrointestinal CD -associated strictures are explored in a cohort study [57].

The collective findings underscore the dynamic nature of endoscopic interventions, emphasizing not only their feasibility and safety but also their potential to impact the quality of life for individuals with CD. The discussion delves into the nuanced aspects of each study, addressing the complexities of patient selection, procedural nuances, and the broader implications for the evolving landscape of IBD management. In conclusion, this review not only consolidates current knowledge but also stimulates further research to refine and integrate endoscopic approaches seamlessly into routine clinical practice for improved patient outcomes in the realm of IBD.

*Role of Colonoscopy* 

The exploration of colonoscopic interventions in the management of IBD unveils a spectrum of studies contributing to the optimization of diagnostic and therapeutic procedures. In the randomized, single-center trial of water-aided colonoscopy, the focus is on enhancing mucosal visualization and patient tolerance. The study provides valuable insights into the potential benefits of incorporating water-assisted techniques, potentially improving procedural outcomes for IBD patients undergoing colonoscopy. In a parallel endeavor, the double-blind, randomized, single-center trial on carbon dioxide insufflation during colonoscopy delves into the comparison between carbon dioxide and traditional air insufflation. The findings bear significance for the minimization of patient discomfort and the enhancement of overall procedural experience in the context of IBD. The combined results highlight the ever-changing characteristics of endoscopic treatments, stressing not only their practicality and lack of risk but also their ability to influence the well-being of persons with Crohn’s disease. Research indicates that CO2 insufflation, when compared to air, effectively reduces stomach discomfort, bloating, and flatulence ratings for at least three hours following colonoscopy in individuals with IBD. This method achieves similar benefits during the procedure itself. A lower level of ferritin was found to be linked to a higher chance of developing IBD in a study that followed participants over time. Iron deficiency was more prevalent in healthy males who later had IBD compared to matched controls, but it was not observed in women. Water-aided colonoscopy is more effective than air insufflation in reducing discomfort in sedated individuals with inflammatory bowel illness who require immediate attention. The procedure also yields similar results in terms of outcomes [58-60].

This study presents a non-invasive approach to assess disease activity after surgery, offering a potential avenue for timely intervention in the postoperative phase. Confocal laser endomicroscopy’s (CLE) pilot study explores its application for the differential diagnosis of UC and CD [61]. The study introduces the prospect of a more accurate diagnostic tool, which could potentially refine treatment strategies based on a precise understanding of the underlying disease. A study conducted by Tontini et al. reveals that CLE has the ability to observe certain microscopic characteristics that are commonly employed in traditional histopathology to distinguish between UC and CD [62]. Nevertheless, due to the restricted ability of CLE to reach deep into tissues, it was not possible to observe submucosal details or granulomas. The novel scoring system has the potential to enable in vivo diagnosis of UC or CD [62]. The study underscores the evolving landscape of novel interventions that can be administered via colonoscopy for the management of IBD. The randomized trial comparing high-definition colonoscopy techniques addresses the critical aspect of lesion detection during IBD surveillance colonoscopy [63].

This study is poised to influence future surveillance strategies, emphasizing the importance of optimizing colonoscopy protocols for improved neoplastic lesion detection. As future trends, these studies collectively suggest a trajectory toward refined colonoscopic techniques for IBD management. The incorporation of advanced imaging modalities, innovative insufflation methods, and the ongoing exploration of non-invasive markers indicate a dynamic evolution within the field. These findings lay the groundwork for enhancing diagnostic accuracy, therapeutic efficacy, and overall patient outcomes in the continually advancing realm of minimally invasive approaches to IBD [64-69].

These findings collectively suggest that minimally invasive techniques offer significant benefits in the management of IBD. Further research, including randomized trials and systematic reviews, is needed to solidify the advantages and address the limitations of these approaches.

Discussion

The findings from our literature review provide a comprehensive overview of the benefits and limitations of minimally invasive techniques in the surgical management of IBD. These techniques, including conventional multiport laparoscopy, single-incision or single-port laparoscopy, and robotic or robot-assisted surgery, have been shown to offer various advantages over traditional open surgery, contributing to improved patient outcomes. In this discussion, we interpret these results and explore their implications for clinical practice.

MIS has gained prominence in the management of IBD, particularly for CD and UC, due to its potential benefits over traditional open surgery. These benefits include reduced postoperative pain, shorter hospital stays, faster recovery times, and improved cosmetic outcomes. This discussion explores the current state of MIS in IBD management, highlights significant advancements, and examines future trends.

Current State of MIS in IBD Management

Laparoscopic surgery: Laparoscopic surgery has been extensively adopted in the management of IBD. Multiple studies have demonstrated its efficacy and safety in both elective and emergency settings. For instance, laparoscopic ileocolic resection for CD is associated with lower morbidity compared to open surgery [66]. Similarly, laparoscopic total proctocolectomy with IPAA for UC results in better fertility outcomes compared to open surgery [64,70-72].

Single-incision laparoscopic surgery: SILS is an evolving technique that further reduces the invasiveness of traditional laparoscopy. Studies have shown that SILS can be feasibly and safely performed for IBD surgeries. There are successful outcomes with SILS for ileocolic resection in CD patients, highlighting reduced postoperative pain and quicker recovery times compared to conventional multiport laparoscopy [73]. Additionally, this article also demonstrates the effectiveness of SILS in pediatric patients with CD [50].

Surgical management of IBD: Even though medical management is the first line of treatment for patients with IBD, the majority of patients may need to undergo surgery at some instance in the course of the disease [74-77]. The main indications for surgery in UC are a refractory course of the disease, emergency indications for severe colitis refractory to medical treatment, and risk of malignant transformation. Total proctocolectomy followed by ileal J pouch with IPAA provides a permanent cure for patients with UC, but the diverting stoma thus significantly affects the quality of life of the patients [78]. Recent advances include performing an LRP without diverting stoma, which reduces the complications associated with open surgery and also improves the postoperative quality of life [79].

A study conducted by Polle et al. reveals that open restorative proctocolectomy (ORP) has a detrimental effect on body image and cosmesis when compared to LRP. The two treatments yield comparable results in terms of functional outcome, quality of life, and morbidity. The benefits of a durable, enhanced body image and cosmesis for this relatively youthful demographic may outweigh the extended surgical durations and increased expenses, especially for females [80]. MIS leads to better patient satisfaction in terms of cosmesis as it may lead to an improved body image as compared to an open surgery [81]. Many benefits are highlighted in studies regarding single-incision or single-port laparoscopic surgery compared to conventional multiport laparoscopic surgery. They are reduced length of stay, lower estimated blood loss, shorter operative time, reduced postoperative pain scores, and reduced need for analgesia post-surgery [82-86]. MIS, including SILS and robot-assisted surgery, is gaining importance in the pediatric population due to better outcomes, such as shorter hospital stays and lower complication rates [87-92]. MIS should be preferred in the pediatric population as multiple surgical scars may have a negative impact on the mental health of the children and may lead to low self-esteem.

Robotic surgery: In recent years, robot-assisted surgery has been performed in the surgical management of IBD. It consists of a surgeon who sits at a console that is detached from the patient with robotic micro instruments and 1080-pixel high-definition 3D cameras. It is possible to create 3D models of the organs in the preoperative period, which helps in better preparation before surgery. It helps augment the surgeon’s dexterity to achieve high precision and accuracy during surgery. It enables telesurgery, which eliminates the need for the physical presence of a surgeon. The disadvantages include high costs, advanced communication technology, and longer operative time [93-95]. Surgeons require additional training and have a steep learning curve as depth perception and tactile perception are lost. Robotic surgery shows similar rates of postoperative complications, readmission, and reoperation as compared to conventional laparoscopic techniques. A study by Raskin et al. demonstrated longer operative time, shorter hospital stays, and a lower rate of 30-day postoperative complications [96].

Recent advances in colorectal robotic surgery include complete robotic surgery with intra-abdominal anastomoses [97]. There is a lack of significant benefit of robotic surgery over laparoscopic surgery, but robotic surgery is beneficial compared to open surgery [98]. Robotic-assisted surgery is increasingly being utilized for IBD management, providing enhanced dexterity and precision. Robotic total proctocolectomy and IPAA have shown comparable short-term outcomes to laparoscopic approaches, with potential benefits in complex cases [70]. Articles report successful robotic ileocolic resections with intracorporeal anastomosis for CD, suggesting the technique’s safety and efficacy [94].

Natural orifice specimen extraction eliminates the need for additional incisions for extraction of resected specimens. This procedure can be performed in patients with UC undergoing colonic resection as well as patients with CD undergoing ileocolic resection and colectomies [99,100]. 

Significant Advancements in MIS for IBD

Enhanced recovery pathways: The implementation of Enhanced Recovery After Surgery (ERAS) pathway protocols has further improved outcomes for IBD patients undergoing MIS. These pathways focus on multimodal perioperative care, reducing surgical stress and enhancing postoperative recovery [101]. An effective ERAS protocol is proven to be beneficial in colorectal surgery with a shorter hospital stay [101] and decreased morbidity and mortality [102,103]. Studies have demonstrated that ERAS protocols combined with laparoscopic surgery significantly reduce hospital stays and improve patient outcomes [75].

Conventional multiport laparoscopy: Conventional multiport laparoscopy is currently the most popular minimally invasive technique for the surgical management of IBD. Its widespread can be attributed to multiple factors, including the fact that it was the earliest minimally invasive surgical technique to be used, and the first laparoscopic resection for IBD was performed in 1992 [104]. The advantages of laparoscopy over open approach have been demonstrated by multiple studies. The current guideline on the surgical management of CD by The American Society of Colon and Rectal Surgeons (ASCRS) recommends the use of a minimally invasive approach [105]. Similarly, for UC, MIS can be considered when the expertise is available as it leads to lower adhesion and increased fertility preservation according to guidelines on the surgical management of UC by ASCRS [106].

Technological innovations: Technological advancements such as CLE and high-definition colonoscopy have enhanced the diagnostic and therapeutic capabilities of MIS. CLE allows real-time histological examination during endoscopy, aiding in the differential diagnosis of UC and CD [61]. High-definition dye spraying and electronic virtual chromoendoscopy improve the detection of neoplastic lesions during IBD surveillance [63].

Prophylactic and therapeutic endoscopic interventions: The comprehensive literature review on endoscopic interventions within MIS for IBD reveals a multifaceted landscape of evolving approaches and promising outcomes.

Endoscopic balloon dilation and stricturoplasty are minimally invasive alternatives for managing strictures in CD. These interventions have shown favorable outcomes, particularly when combined with immunomodulatory therapy [55]. Additionally, the use of probiotics such as VSL#3 has demonstrated anti-inflammatory effects and reduced endoscopic recurrence after CD surgery [54].

In summary, this review not only compiles current understanding but also promotes additional research to enhance and incorporate endoscopic methods into standard clinical practice, ultimately aiming to improve patient outcomes in the treatment of IBD.

Future Trends in MIS for IBD

Integration of artificial intelligence (AI): AI is expected to revolutionize MIS in IBD by enhancing diagnostic accuracy, predicting surgical outcomes, and personalizing treatment plans. Machine learning algorithms can assist in identifying subtle mucosal changes during endoscopy, improving early detection and intervention.

Expansion of robotic surgery: The adoption of robotic-assisted techniques is likely to increase, driven by ongoing technological improvements and growing surgeon experience. Future studies should focus on long-term outcomes and the cost-effectiveness of robotic surgery compared to traditional MIS approaches.

Development of next-generation surgical instruments: The continuous evolution of surgical instruments, including flexible and miniaturized devices, will further enhance the precision and safety of MIS. Innovations such as single-port robotic platforms and natural orifice transluminal endoscopic surgery (NOTES) hold promise for reducing the invasiveness of IBD surgeries.

Enhanced biologic and targeted therapies: The integration of advanced biologic and targeted therapies with MIS could improve surgical outcomes and reduce recurrence rates. Personalized medicine, guided by genetic and molecular profiling, will enable tailored treatment strategies, optimizing both medical and surgical management of IBD.

In conclusion, MIS has significantly advanced the management of IBD, offering numerous benefits over traditional surgery. Ongoing research and technological innovations will continue to enhance the efficacy and safety of MIS, improving the quality of life for IBD patients.

Future directions

Although there are multiple options available for surgical management of IBD using MIS, only conventional multiport laparoscopy has been able to gain widespread acceptance, as there is a consistent demonstration of its advantages without a disproportionate increase in costs associated with the surgery. However, this does not invalidate the utility of other techniques as evidence of their advantages could emerge with increased use of such techniques in the coming years. Robotic surgery, in particular, is being investigated further in order to determine the scope of its utility in various surgeries for IBD. There has been an increasing focus on reducing the cost of robotic surgeries in order to make them more affordable for patients so that more evidence can be generated about their advantages after they gain widespread usage.

The steep learning curve associated with robotic surgeries is being addressed so that young surgeons can adopt this technique from early on in their careers. Moreover, future investigations into the use of robotic surgery will focus on outcomes that are clinically relevant to the patients. The role of endoscopy in treating IBD is being further explored in order to discover new avenues for its usage. The usage of endoscopy as an adjunct to laparoscopy is also being explored. Single-incision surgery, natural orifice specimen extraction, and transanal MIS (TAMIS) are being further explored as options for the treatment of varying presentations of IBD. The increased usage of these techniques, with evidence being generated in its favor, could result in these techniques being incorporated into standard practice guidelines in the future.

A significant impact of the adoption of these techniques will be on the pediatric population, as reduced psychological impact of the surgery will lead to overall improved quality of life and better self-esteem. As the course of IBD in children can be challenging, any effort made to incorporate MIS as part of treatment protocol in the surgical management of IBD in children should be encouraged. MIS will not just be used for treatment but also for diagnosis, surveillance, and treatment of complications of IBD. This would ensure higher rates of compliance and earlier detection of complications, such as dysplasia, strictures, and recurrence. Timely intervention would increase the overall quality of life of patients suffering from this chronic and complex disease.

## Conclusions

IBD is a chronic inflammatory disorder affecting the gastrointestinal tract. Minimally invasive techniques like conventional multiport laparoscopy, single-incision laparoscopy, robot-assisted surgery, endoscopy, and colonoscopy are more effective than traditional open surgical approaches in terms of reduced postoperative pain, shorter hospital stays, faster recovery times, and improved cosmetic outcomes. MIS techniques have shown comparable or superior surgical outcomes, including lower rates of postoperative complications and better long-term disease control. However, challenges such as optimizing surgical strategies and integrating laparoscopic techniques with enhanced recovery pathways remain. In pediatric patients, MIS techniques play a crucial role, offering better outcomes and improved quality of life. Endoscopic interventions, such as laparoendoscopic single-site surgery and endoscopic balloon dilation, show promise in treating IBD-related strictures and reducing postoperative complications. Colonoscopic interventions, like water-aided colonoscopy and fecal calprotectin measurement, offer non-invasive methods for monitoring disease activity and detecting recurrence. However, challenges such as cost, limited accessibility to specialized centers, and the learning curve associated with newer technologies remain important considerations.

## References

[1] Rubin DT, Ananthakrishnan AN, Siegel CA, Sauer BG, Long MD (2019). ACG clinical guideline: ulcerative colitis in adults. Am J Gastroenterol.

[2] Heller F, Florian P, Bojarski C (2005). Interleukin-13 is the key effector Th2 cytokine in ulcerative colitis that affects epithelial tight junctions, apoptosis, and cell restitution. Gastroenterology.

[3] Gajendran M, Loganathan P, Jimenez G (2019). A comprehensive review and update on ulcerative colitis. Dis Mon.

[4] Bernstein CN, Rawsthorne P, Cheang M, Blanchard JF (2006). A population-based case control study of potential risk factors for IBD. Am J Gastroenterol.

[5] Petagna L, Antonelli A, Ganini C (2020). Pathophysiology of Crohn's disease inflammation and recurrence. Biol Direct.

[6] Cheifetz AS (2013). Management of active Crohn disease. JAMA.

[7] Paul G, Schäffler A, Neumeier M (2006). Profiling adipocytokine secretion from creeping fat in Crohn's disease. Inflamm Bowel Dis.

[8] Ng SC, Shi HY, Hamidi N (2017). Worldwide incidence and prevalence of inflammatory bowel disease in the 21st century: a systematic review of population-based studies. Lancet.

[9] Loftus EV Jr (2004). Clinical epidemiology of inflammatory bowel disease: incidence, prevalence, and environmental influences. Gastroenterology.

[10] Benchimol EI, Fortinsky KJ, Gozdyra P, Van den Heuvel M, Van Limbergen J, Griffiths AM (2011). Epidemiology of pediatric inflammatory bowel disease: a systematic review of international trends. Inflamm Bowel Dis.

[11] Gasparetto M, Guariso G (2013). Highlights in IBD epidemiology and its natural history in the paediatric age. Gastroenterol Res Pract.

[12] Travis SP, Stange EF, Lémann M (2008). European evidence-based consensus on the management of ulcerative colitis: current management. J Crohns Colitis.

[13] Filippi J, Allen PB, Hébuterne X, Peyrin-Biroulet L (2011). Does anti-TNF therapy reduce the requirement for surgery in ulcerative colitis? A systematic review. Curr Drug Targets.

[14] Targownik LE, Singh H, Nugent Z, Bernstein CN (2012). The epidemiology of colectomy in ulcerative colitis: results from a population-based cohort. Am J Gastroenterol.

[15] Sica GS, Biancone L (2013). Surgery for inflammatory bowel disease in the era of laparoscopy. World J Gastroenterol.

[16] Maggiori L, Panis Y (2013). Surgical management of IBD--from an open to a laparoscopic approach. Nat Rev Gastroenterol Hepatol.

[17] Andrews HA, Lewis P, Allan RN (1989). Prognosis after surgery for colonic Crohn's disease. Br J Surg.

[18] Shrestha B (2016). Minimally invasive surgery for inflammatory bowel disease: current perspectives. World J Gastrointest Pharmacol Ther.

[19] Karayiannakis AJ, Makri GG, Mantzioka A, Karousos D, Karatzas G (1996). Postoperative pulmonary function after laparoscopic and open cholecystectomy. Br J Anaesth.

[20] Scarpa M, Di Cristofaro L, Cortinovis M (2013). Minimally invasive surgery for colorectal cancer: quality of life and satisfaction with care in elderly patients. Surg Endosc.

[21] Palep JH (2009). Robotic assisted minimally invasive surgery. J Minim Access Surg.

[22] Holubar SD, Wolff BG (2009). Advances in surgical approaches to Crohn's disease: minimally invasive surgery and biologic therapy. Expert Rev Clin Immunol.

[23] Courtney ED, Brennan M, Noble-Jamieson G, Heuschkel R, Davies RJ (2011). Laparoscopic adult colorectal surgeon and adolescents with inflammatory bowel disease: a safe combination?. Int J Colorectal Dis.

[24] Eshuis EJ, Slors JF, Stokkers PC (2010). Long-term outcomes following laparoscopically assisted versus open ileocolic resection for Crohn's disease. Br J Surg.

[25] Hor T, Zalinski S, Lefevre JH, Shields C, Attal E, Tiret E, Parc Y (2012). Feasibility of laparoscopic restorative proctocolectomy without diverting stoma. Dig Liver Dis.

[26] Hull TL, Joyce MR, Geisler DP, Coffey JC (2012). Adhesions after laparoscopic and open ileal pouch-anal anastomosis surgery for ulcerative colitis. Br J Surg.

[27] Bartels SA, DʼHoore A, Cuesta MA, Bensdorp AJ, Lucas C, Bemelman WA (2012). Significantly increased pregnancy rates after laparoscopic restorative proctocolectomy: a cross-sectional study. Ann Surg.

[28] Maartense S, Dunker MS, Slors JF (2006). Laparoscopic-assisted versus open ileocolic resection for Crohn's disease: a randomized trial. Ann Surg.

[29] Palanivelu C, Jani K, Sendhilkumar K, Parthasarathi R, Senthilnathan P, Maheshkumar G (2008). Laparoscopic restorative total proctocolectomy with ileal pouch anal anastomosis for familial adenomatous polyposis. JSLS.

[30] Lim MH, Lord AR, Simms LA (2021). Ileal pouch-anal anastomosis for ulcerative colitis: an Australian institution’s experience. Ann Coloproctol.

[31] Miller AT, Berian JR, Rubin M, Hurst RD, Fichera A, Umanskiy K (2012). Robotic-assisted proctectomy for inflammatory bowel disease: a case-matched comparison of laparoscopic and robotic technique. J Gastrointest Surg.

[32] Celentano V, Pellino G, Rottoli M, Colombo F, Sampietro G, Spinelli A, Selvaggi F (2021). Single-incision laparoscopic surgery (SILS) for the treatment of ileocolonic Crohn's disease: a propensity score-matched analysis. Int J Colorectal Dis.

[33] Gardenbroek TJ, Verlaan T, Tanis PJ, Ponsioen CY, D'Haens GR, Buskens CJ, Bemelman WA (2013). Single-port versus multiport laparoscopic ileocecal resection for Crohn's disease. J Crohns Colitis.

[34] Carvello M, de Groof EJ, de Buck van Overstraeten A (2018). Single port laparoscopic ileocaecal resection for Crohn's disease: a multicentre comparison with multi-port laparoscopy. Colorectal Dis.

[35] Li W, Rencuzogullari A, Costedio M, Benlice C, Kessler H, Stocchi L, Gorgun E (2019). Outcome comparison of single-port versus multiport versus under direct view completion proctectomy with ileal-pouch anal anastomosis for patients with ulcerative colitis. Surg Laparosc Endosc Percutan Tech.

[36] Burke J, Toomey D, Reilly F, Cahill R (2020). Single access laparoscopic total colectomy for severe refractory ulcerative colitis. World J Gastroenterol.

[37] Raskin ER, Gorrepati ML, Mehendale S, Gaertner WB (2019). Robotic-assisted ileocolic resection for Crohn's disease: outcomes from an early national experience. J Robot Surg.

[38] Opoku D, Hart A, Thompson DT (2022). Equivalency of short-term perioperative outcomes after open, laparoscopic, and robotic ileal pouch anal anastomosis. Does procedure complexity override operative approach?. Surg Open Sci.

[39] Anderson M, Lynn P, Aydinli HH, Schwartzberg D, Bernstein M, Grucela A (2020). Early experience with urgent robotic subtotal colectomy for severe acute ulcerative colitis has comparable perioperative outcomes to laparoscopic surgery. J Robot Surg.

[40] Aydinli HH, Anderson M, Hambrecht A, Bernstein MA, Grucela AL (2021). Robotic ileocolic resection with intracorporeal anastomosis for Crohn's disease. J Robot Surg.

[41] Lightner AL, Grass F, McKenna NP (2019). Short-term postoperative outcomes following robotic versus laparoscopic ileal pouch-anal anastomosis are equivalent. Tech Coloproctol.

[42] Miller AT, Berian JR, Rubin M, Hurst RD, Fichera A, Umanskiy K (2018). The role of robotics in colorectal surgery. BMJ.

[43] Rencuzogullari A, Gorgun E, Costedio M, Aytac E, Kessler H, Abbas MA, Remzi FH (2016). Case-matched comparison of robotic versus laparoscopic proctectomy for inflammatory bowel disease. Surg Laparosc Endosc Percutan Tech.

[44] Rencuzogullari A, Benlice C, Costedio M, Kessler H, Ashburn J, Stocchi L, Gorgun E (2016). Mo1767 comparison of robotic versus single-port laparoscopic completion proctectomy with IPAA for ulcerative colitis. Gastroenterology.

[45] Gebhardt JM, Werner N, Stroux A (2022). Robotic-assisted versus laparoscopic proctectomy with ileal pouch-anal anastomosis for ulcerative colitis: an analysis of clinical and financial outcomes from a tertiary referral center. J Clin Med.

[46] Linden BC, Bairdain S, Zurakowski D, Shamberger RC, Lillehei CW (2013). Comparison of laparoscopic-assisted and open total proctocolectomy and ileal pouch anal anastomosis in children and adolescents. J Pediatr Surg.

[47] Willobee BA, Nguyen JA, Ferrantella A (2021). A retrospective comparison of outcomes for open vs. laparoscopic surgical techniques in pediatric ulcerative colitis. Transl Gastroenterol Hepatol.

[48] Quiroz HJ, Perez EA, El Tawil RA (2020). Open versus laparoscopic right Hemicolectomies in pediatric patients with Crohn’s disease. J Laparoendosc Adv Surg Tech A.

[49] Huntington JT, Boomer LA, Pepper VK, Diefenbach KA, Dotson JL, Nwomeh BC (2016). Single-incision laparoscopic surgery (SILS) for children with Crohn's disease. Pediatr Surg Int.

[50] Sharp NE, Thomas P, St Peter SD (2014). Single-incision laparoscopic ileocecectomy in children with Crohn's disease. J Laparoendosc Adv Surg Tech A.

[51] Romeo C, Di Fabrizio D, Impellizzeri P (2022). Laparoscopic robotic-assisted restorative proctocolectomy and ileal J-pouch-anorectal anastomosis in children. Pediatr Surg Int.

[52] Gash KJ, Goede AC, Chambers W, Greenslade GL, Dixon AR (2011). Laparoendoscopic single-site surgery is feasible in complex colorectal resections and could enable day case colectomy. Surg Endosc.

[53] Wu XR, Mukewar S, Kiran RP, Remzi FH, Shen B (2013). Surgical stricturoplasty in the treatment of ileal pouch strictures. J Gastrointest Surg.

[54] Fedorak RN, Feagan BG, Hotte N (2015). The probiotic VSL#3 has anti-inflammatory effects and could reduce endoscopic recurrence after surgery for Crohn's disease. Clin Gastroenterol Hepatol.

[55] Honzawa Y, Nakase H, Matsuura M (2013). Prior use of immunomodulatory drugs improves the clinical outcome of endoscopic balloon dilation for intestinal stricture in patients with Crohn's disease. Dig Endosc.

[56] Karahan Ö, Sevinç B (2021). Endoscopic approaches for prophylactic purposes. Prophylactic Surgery.

[57] Singh A, Agrawal N, Kurada S (2017). Efficacy, safety, and long-term outcome of serial endoscopic balloon dilation for upper gastrointestinal Crohn’s disease-associated strictures—a cohort study. J Crohns Colitis.

[58] Falt P, Šmajstrla V, Fojtík P, Urban O, Hill M (2015). Water-aided colonoscopy in inflammatory bowel disease patients-a randomised, single-centre trial. J Crohns Colitis.

[59] Falt P, Šmajstrla V, Fojtík P, Hill M, Urban O (2017). Carbon dioxide insufflation during colonoscopy in inflammatory bowel disease patients: a double-blind, randomized, single-center trial. Eur J Gastroenterol Hepatol.

[60] Widbom L, Ekblom K, Karling P, Hultdin J (2020). Patients developing inflammatory bowel disease have iron deficiency and lower plasma ferritin years before diagnosis: a nested case-control study. Eur J Gastroenterol Hepatol.

[61] Wright EK, Kamm MA, De Cruz P (2015). Measurement of fecal calprotectin improves monitoring and detection of recurrence of Crohn's disease after surgery. Gastroenterology.

[62] Tontini GE, Mudter J, Vieth M (2015). Confocal laser endomicroscopy for the differential diagnosis of ulcerative colitis and Crohn's disease: a pilot study. Endoscopy.

[63] Damman CJ, Brittnacher MJ, Westerhoff M (2015). Low level engraftment and improvement following a single colonoscopic administration of fecal microbiota to patients with ulcerative colitis. PLoS One.

[64] Iacucci M, Kaplan GG, Panaccione R (2018). A randomized trial comparing high definition colonoscopy alone with high definition dye spraying and electronic virtual chromoendoscopy for detection of colonic neoplastic lesions during IBD surveillance colonoscopy. Am J Gastroenterol.

[65] Beyer-Berjot L, Maggiori L, Birnbaum D, Lefevre JH, Berdah S, Panis Y (2013). A total laparoscopic approach reduces the infertility rate after ileal pouch-anal anastomosis: a 2-center study. Ann Surg.

[66] Ponder A, Long MD (2013). A clinical review of recent findings in the epidemiology of inflammatory bowel disease. Clin Epidemiol.

[67] Reynolds IS, Doogan KL, Ryan ÉJ, Hechtl D, Lecot FP, Arya S, Martin ST (2021). Surgical strategies to reduce postoperative recurrence of Crohn’s disease after ileocolic resection. Front Surg.

[68] Greenstein AJ, Romanoff AM, Moskowitz AJ, Sosunov EA, Khaitov S, Egorova NN (2013). Payer status and access to laparoscopic subtotal colectomy for ulcerative colitis. Dis Colon Rectum.

[69] Piraka C, Shah RJ, Fukami N, Chathadi KV, Chen YK (2009). EUS-guided transesophageal, transgastric, and transcolonic drainage of intra-abdominal fluid collections and abscesses. Gastrointest Endosc.

[70] Holder-Murray J, Zoccali M, Hurst RD, Umanskiy K, Rubin M, Fichera A (2012). Totally laparoscopic total proctocolectomy: a safe alternative to open surgery in inflammatory bowel disease. Inflamm Bowel Dis.

[71] Krane MK, Allaix ME, Zoccali M (2013). Preoperative infliximab therapy does not increase morbidity and mortality after laparoscopic resection for inflammatory bowel disease. Dis Colon Rectum.

[72] Miller AT, Berian JR, Rubin M, Hurst RD, Fichera A, Umanskiy K (2011). Robotic-assisted proctectomy for inflammatory bowel disease: a case matched comparative study of laparoscopic and robotic-assisted restorative and completion proctectomy. Gastroenterology.

[73] Rijcken E, Mennigen R, Argyris I, Senninger N, Bruewer M (2012). Single-incision laparoscopic surgery for ileocolic resection in Crohn's disease. Dis Colon Rectum.

[74] Meima-van Praag EM, Buskens CJ, Hompes R, Bemelman WA (2021). Surgical management of Crohn's disease: a state of the art review. Int J Colorectal Dis.

[75] Spinelli A, Bazzi P, Sacchi M (2013). Short-term outcomes of laparoscopy combined with enhanced recovery pathway after ileocecal resection for Crohn's disease: a case-matched analysis. J Gastrointest Surg.

[76] Peyrin-Biroulet L, Lémann M (2011). Review article: remission rates achievable by current therapies for inflammatory bowel disease. Aliment Pharmacol Ther.

[77] Samuel S, Ingle SB, Dhillon S (2013). Cumulative incidence and risk factors for hospitalization and surgery in a population-based cohort of ulcerative colitis. Inflamm Bowel Dis.

[78] Kolho KL, Nikkonen A, Merras-Salmio L, Molander P (2024). The need for surgery in pediatric patients with inflammatory bowel disease treated with biologicals. Int J Colorectal Dis.

[79] Alabdulrahman M, Stuart L, Smith E, O'Connell PR (2023). Recurrent pouch volvulus following ileoanal J-pouch anastomosis: a case report. Cureus.

[80] Polle SW, Dunker MS, Slors JF, Sprangers MA, Cuesta MA, Gouma DJ, Bemelman WA (2007). Body image, cosmesis, quality of life, and functional outcome of hand-assisted laparoscopic versus open restorative proctocolectomy: long-term results of a randomized trial. Surg Endosc.

[81] Fichera A, Zoccali M, Felice C, Rubin DT (2011). Total abdominal colectomy for refractory ulcerative colitis: surgical treatment in evolution. J Gastrointest Surg.

[82] Baek SJ, Lightner AL, Boostrom SY (2017). Functional outcomes following laparoscopic ileal pouch-anal anastomosis in patients with chronic ulcerative colitis: long-term follow-up of a case-matched study. J Gastrointest Surg.

[83] Bhattacharya P, Hussain MI, Zaman S (2023). Single-incision versus multi-port laparoscopic ileocolic resections for Crohn's disease: systematic review and meta-analysis. J Minim Access Surg.

[84] Babunashvili EL, Son DY, Buyanova SN (2023). Outcomes of laparotomic myomectomy during pregnancy for symptomatic uterine fibroids: a prospective cohort study. J Clin Med.

[85] Dotlacil V, Lerchova T, Coufal S (2023). Comparison of laparoscopic and open ileocecal resection for Crohn's disease in children. Pediatr Surg Int.

[86] Liu W, Zhou W (2023). Minimally invasive surgery in Crohn's disease: state-of-the-art review. Front Surg.

[87] Barmparas G, Branco BC, Schnüriger B, Lam L, Inaba K, Demetriades D (2010). The incidence and risk factors of post-laparotomy adhesive small bowel obstruction. J Gastrointest Surg.

[88] Tan Tanny SP, Yoo M, Hutson JM, Langer JC, King SK (2019). Current surgical practice in pediatric ulcerative colitis: a systematic review. J Pediatr Surg.

[89] Schaefer ME, Machan JT, Kawatu D (2010). Factors that determine risk for surgery in pediatric patients with Crohn's disease. Clin Gastroenterol Hepatol.

[90] Panteleimonitis S, Al-Dhaheri M, Harper M, Amer I, Ahmed AA, Nada MA, Parvaiz A (2023). Short-term outcomes in robotic vs laparoscopic ileal pouch-anal anastomosis surgery: a propensity score match study. Langenbecks Arch Surg.

[91] Olson CH, Bedros N, Hakiman H, Araghizadeh FY (2014). Single-site laparoscopic surgery for inflammatory bowel disease. JSLS.

[92] Garey CL, Laituri CA, Ostlie DJ, St Peter SD (2010). A review of single site minimally invasive surgery in infants and children. Pediatr Surg Int.

[93] Zaman S, Mohamedahmed AY, Abdelrahman W (2024). Minimally invasive surgery for inflammatory bowel disease: a systematic review and meta-analysis of robotic versus laparoscopic surgical techniques. J Crohns Colitis.

[94] Ruurda JP, Draaisma WA, van Hillegersberg R (2005). Robot-assisted endoscopic surgery: a four-year single-center experience. Dig Surg.

[95] White I, Jenkins JT, Coomber R, Clark SK, Phillips RK, Kennedy RH (2014). Outcomes of laparoscopic and open restorative proctocolectomy. Br J Surg.

[96] Calini G, Abdalla S, Abd El Aziz MA, Merchea A, Larson DW, Behm KT (2023). Ileocolic resection for Crohn's disease: robotic intracorporeal compared to laparoscopic extracorporeal anastomosis. J Robot Surg.

[97] Lujan HJ, Molano A, Burgos A, Rivera B, Plasencia G (2015). Robotic right colectomy with intracorporeal anastomosis: experience with 52 consecutive cases. J Laparoendosc Adv Surg Tech A.

[98] Kim CW, Kim CH, Baik SH (2014). Outcomes of robotic-assisted colorectal surgery compared with laparoscopic and open surgery: a systematic review. J Gastrointest Surg.

[99] Gardenbroek TJ, Eshuis EJ, van Acker GJ, Tanis PJ, Bemelman WA (2012). Alternative specimen extraction techniques after laparoscopic emergency colectomy in inflammatory bowel disease. Surg Endosc.

[100] Eshuis EJ, Voermans RP, Stokkers PC, van Berge Henegouwen MI, Fockens P, Bemelman WA (2010). Laparoscopic resection with transcolonic specimen extraction for ileocaecal Crohn's disease. Br J Surg.

[101] Lovely JK, Maxson PM, Jacob AK (2012). Case-matched series of enhanced versus standard recovery pathway in minimally invasive colorectal surgery. Br J Surg.

[102] Spanjersberg WR, Reurings J, Keus F, van Laarhoven CJ (2011). Fast track surgery versus conventional recovery strategies for colorectal surgery. Cochrane Database Syst Rev.

[103] Varadhan KK, Neal KR, Dejong CH, Fearon KC, Ljungqvist O, Lobo DN (2010). The enhanced recovery after surgery (ERAS) pathway for patients undergoing major elective open colorectal surgery: a meta-analysis of randomized controlled trials. Clin Nutr.

[104] Sardinha TC, Wexner SD (1998). Laparoscopy for inflammatory bowel disease: pros and cons. World J Surg.

[105] Lightner AL, Vogel JD, Carmichael JC (2020). The American Society of Colon and Rectal Surgeons clinical practice guidelines for the surgical management of Crohn’s disease. Dis Colon Rectum.

[106] Ross H, Steele SR, Varma M, Dykes S, Cima R, Buie WD, Rafferty J (2014). Practice parameters for the surgical treatment of ulcerative colitis. Dis Colon Rectum.

